# Leaching Characteristics of Heavy Metals in the Baghouse Filter Dust from Direct-Fired Thermal Desorption of Contaminated Soil

**DOI:** 10.3390/ijerph192416504

**Published:** 2022-12-08

**Authors:** Panpan Wang, Yunzhe Cao, Bin Yang, Huilong Luo, Tian Liang, Jingjing Yu, Aizhong Ding, Lina Wang, Huiying Li, Hanlin Cao, Fujun Ma, Qingbao Gu, Fasheng Li

**Affiliations:** 1 College of Water Sciences, Beijing Normal University, Beijing 100875, China; 2 State Key Laboratory of Environmental Criteria and Risk Assessment, Chinese Research Academy of Environmental Sciences, Beijing 100012, China; 3 Technical Centre for Soil, Agriculture and Rural Ecology and Environment, Ministry of Ecology and Environment, Beijing 100012, China; 4 School of Chemical and Environmental Engineering, China University of Mining and Technology, Beijing 100083, China

**Keywords:** thermal desorption, baghouse filter dust, heavy metal, leaching behavior, soil, environmental effects

## Abstract

After thermal desorption, the total amount of heavy metals (HMs) is enriched in baghouse filter dust. To further understand the related environmental impact, the leaching characteristics under various conditions must be explored. Therefore, this study aimed to examine the leaching characteristics of seven HMs in the dust generated in the direct-fired thermal desorption process and to compare the differences in heavy metal leaching characteristics in the soil before and after thermal desorption. The leaching characteristics and bioaccessibility of seven HMs—arsenic (As), cadmium (Cd), chromium (Cr), copper (Cu), lead (Pb), nickel (Ni), and zinc (Zn)—were analyzed in dust and in soil before and after thermal desorption. The activity of HMs in dust was strong. Therefore, environmental effects and effects on human health should be considered in the treatment of soil and dust after thermal desorption.

## 1. Introduction

Ex-situ thermal desorption has clear advantages in the treatment of organic pollutants in contaminated soil [1]. However, in the thermal desorption treatment of target pollutants, a higher temperature environment also affects the physicochemical properties of soil [2]. During the treatment of soil contaminated with organic compounds through thermal desorption, the transformation of HMs is often overlooked. Therefore, the influence of the entire process of ex-situ direct-fired thermal desorption (Figure S1a,g) on HMs was the focus of this study.

During thermal desorption, changes in HMs in soil are affected primarily by the target temperature, heating time, and properties of the metals [1,3]. Studies have shown that thermal desorption technology performs well in removing polycyclic aromatic hydrocarbons (PAHs) from coking plant soil, but the differences in the total amounts and chemical species of HMs before and after thermal desorption are not significant [4]. Some studies have indicated that effect of thermal desorption on the total HM amount is unclear but has a substantial influence on the activity of HMs [5,6]. PAHs are target pollutants in contaminated soil. After thermal desorption, the changes in the chemical state of the HMs in soil lead to enhanced bioaccessibility, and Cd, Ni, and Cr have been found to lead to genotoxicity in organisms [5]. One study has shown that the proportion of the water-soluble and exchangeable fractions of Zn and Fe in the soil decreases after thermal desorption, thus implying that these two HMs are solidified through this process [7].

However, after direct-fired thermal desorption, the final solid waste to be disposed of includes not only the soil at the exit of the rotary kiln but also the dust in the bag dust collector (Figure S1). Nonetheless, little attention has been paid to the forms and leaching characteristics of HMs in this dust.

Previous studies have reported that after the thermal desorption treatment of PAH-contaminated soil, seven HMs are enriched in the baghouse filter dust (Table S1) [8]. However, the pollution characteristics of this dust cannot be assessed on the basis of the total amounts of HMs in the dust. The activity and bioaccessibility of HMs are important indexes for the comprehensive assessment of the environmental pollution characteristics of HMs in dust [9]. Moreover, possible changes in the leaching characteristics of HMs in the dust may result in potential contamination of the soil, groundwater, and surrounding environment, thus ultimately affecting human health [10].

Therefore, a comprehensive evaluation of the leaching characteristics of HMs in dust, including further assessment of the differences in leaching characteristics of HMs in baghouse filter dust, is necessary. In this study, we focused on the leaching characteristics of HMs in baghouse filter dust and compared them with the leaching characteristics of HMs in corresponding soils before and after thermal desorption. Furthermore, we quantified the leaching characteristics of HMs in the potential solid waste (dust) through a multi-dimensional analysis to provide a scientific basis for the management and disposal of the dust generated in ex-situ direct-fired thermal desorption sites, to minimize the possible negative effects of these solid wastes on human health.

## 2. Materials and Methods

### 2.1. Sample Collection and Processing

In this study, contaminated soil was collected from a non-ferrous metal processing plant in northeastern China (Figure S1) with a long production history. This plant was in continuous operation between 1949 and 2007. The pollution characteristics of the soil were assessed. The detailed sample collection and processing steps are shown in Figure S1.

### 2.2. One-Step Batch Leaching Tests

The leaching of seven types of HMs was assessed with the one-step batch leaching test [11]. This test was performed in triplicate on soil, thermal desorption (TD) soil, and dust, at a liquid-to-solid ratio of 10 L:1 kg dry matter (L/S 10) for three types of samples with particle sizes below 4 mm [12]. The samples were agitated in deionized water in 500 mL high-density polyethylene bottles on a rotating device for 24 h, after which they were filtered through 0.45 μm filter membranes. The leachates were stored at 4 °C before analysis. Finally, the concentrations of As, Cd, Cr, Cu, Pb, Ni, and Zn in leachates were determined with ICP-MS (7900, Agilent, Santa Clara, CA, USA).

### 2.3. Toxicity Characteristic Leaching Procedure

A toxicity characteristic leaching procedure (TCLP) [13] was designed to determine the mobility of both organic and inorganic analytes present in the liquid, solid, and multiphasic wastes. Two types of extraction fluids were used. For fluid #1, 5.7 mL acetic acid was added to a volumetric flask and adjusted with deionized water to a 500 mL volume, then transferred to a larger volumetric flask to which 64.3 mL sodium hydroxide solution (1 mol/L) was added, and deionized water was used to adjust the volume to 1 L. For fluid #2, 5.7 mL acetic acid was dissolved in deionized water in a 1 L volumetric flask. The pH of fluid #1 was 4.93 ± 0.05 and that of fluid #2 was 2.88 ± 0.05. The selection of fluid was determined on the basis of preliminary experiments.

Samples were passed through an 8-mesh (approximately 2 mm) sieve, and 5 g was weighed and placed in 100 mL of fluid #1 or fluid #2 for the leaching experiments. Diagonal sampling was kept as far as possible during sampling to decrease error due to the heterogeneity of the soil samples. The liquid-to-solid ratio was 20:1. Solutions were oscillated at room temperature for 18 h at a speed of 30 ± 2 r/min, centrifuged at 3000 r/min for 15 min, and filtered through a 0.45 μm filter membrane with one or two drops of HNO_3_ to adjust the pH to <2.00. Finally, the concentrations of As, Cd, Cu, Cr, Pb, Ni, and Zn were determined with ICP-MS (7900, Agilent, Santa Clara, CA, USA).

### 2.4. Simple Bioaccessibility Extraction Test

The simple bioaccessibility extraction test (SBET), British Geological Survey (BGS), United Kingdom simulates the mobilization of contaminants in the acidic conditions of the stomach. The soil samples used in the experiment were passed through a 60-mesh sieve (approximately 250 µm). The pre-treated soil samples (2 g) were then placed in a 250 mL centrifuge tube. A total of 200 mL of 0.4 mol/L amino-acetic acid-salt acid buffer solution was added, and the pH was adjusted to 1.50 ± 0.05 with concentrated HCl. The lid was closed and tightened at 37.0 °C. The tubes were subjected to an oscillation frequency of 30 ± 2 r/min in a constant temperature water bath oscillator for 1 h. After extraction, the samples were filtered through a 0.45 µm filter membrane, and the solution was acidified to pH < 2.00 with concentrated HNO_3_. The content of heavy metal ions in the solution was determined with ICP-MS (7900, Agilent, Santa Clara, CA, USA) (after extraction, the pH of the extraction system was determined immediately, and if the pH was not in the range of 1.00–2.00, extraction was repeated, until the pH was within this range). The bioaccessibility of HMs in soil was calculated with the following formula:

$$\mathrm{Bioacceccibility}=\frac{C_{B}\times V÷m}{C_{T}}\times 100\%$$

where: *C_B_*: Concentration of a certain heavy metal in the leaching solution, mg/L; *C_T_*: Concentration of a certain heavy metal in the sample, mg/kg; *V*: Volume of the leaching solution, L; and *m*: Quality of the sample, kg.

### 2.5. Observation of Dust Particle Micromorphology

Scanning electron microscopy (SEM; ZEISS Merlin, Oberkochen, Germany) was used to observe the micro-morphology of the samples. Dried and ground samples were evenly dispersed on silicon wafers, and the loose powder was blown off with a rubber suction bulb. Some samples were dispersed in anhydrous ethanol solvent by ultrasound and then dropped onto silicon wafers for drying. The silicon wafers were fixed to conductive double-sided tape. Subsequently, each prepared sample was placed on the sample observation platform.

## 3. Results and Discussion

### 3.1. Leaching Characteristics of Dust and Soil under Different Exposure Conditions

#### 3.1.1. Leaching Behavior of HMs in Nearly Natural Conditions

**Figure 1:**
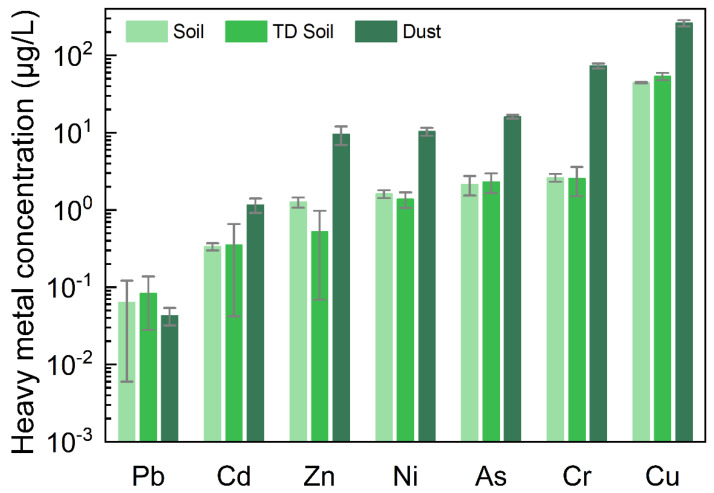
Leaching concentrations of HMs in one-step batch leaching tests (*n* = 3). The content of Pb, Cd, Zn, Ni, As, Cr, and Cu in the samples. The leaching concentrations of HMs, from low to high, were in the following order: Pb, Cd, Zn, Ni, As, Cr, and Cu. Because the concentrations of Pb were near the detection limit, Pb is not included in the discussion of the leaching results in Section 3.1.1. In the dust samples, the leaching concentrations of Cd, Zn, Ni, As, Cr, and Cu were 1.17 μg/L, 9.50 μg/L, 10.35 μg/L, 16.11 μg/L, 73.18 μg/L, and 261.47 μg/L, respectively. The class III groundwater quality standard was used as a reference (Table S2).

Little difference was observed between the heavy metal concentrations in TD soil and the original soil in one-step batch leaching tests [11]. However, except for Pb, the leaching concentrations of the other six HMs in the dust showed significant increases (Figure 1). The leaching concentration of HMs in dust was 3.45–27.86 times that in the soil. The leaching concentrations of HMs, from low to high, were in the following order: Cd < Cu < Ni < As < Zn < Cr. The leaching concentrations of HMs in dust were approximately 3.31–28.55 times those in TD soil. The leaching concentrations of HMs, from low to high, were in the following order: Cd < Cu < As < Ni < Zn < Cr. The concentrations of HMs in dust were clearly higher. Under these extraction conditions, the Cr leaching concentration multiple in dust was highest.

The leaching concentration was compared with the class III groundwater quality standard value (Table S2) [14]. Whereas As had a standard value ≤10 μg/L, the leaching concentration of As in dust reached 16 μg/L. The standard value of Cr (VI) was ≤50 μg/L, whereas the leaching concentration of total Cr in dust reached 73 μg/L. The chemical and physical properties of dust from the baghouse filter differed between the soil and TD soil, and the leaching characteristics of HMs in dust also differed.

In this case, baghouse filter dust refers to backfilling into the original foundation pit disposal. Hence, a possible risk of the dust contaminating soil, groundwater, and the surrounding environment with HMs exists. The experimental results indicated problems when baghouse filter dust mixed with TD soil were disposed of together (Figure S1g). The leaching characteristics of HMs in dust have been found to be altered and to pose likely environmental risks [15].

#### 3.1.2. Leaching Behavior of HMs in Landfill Disposal Scenarios

**Figure 2:**
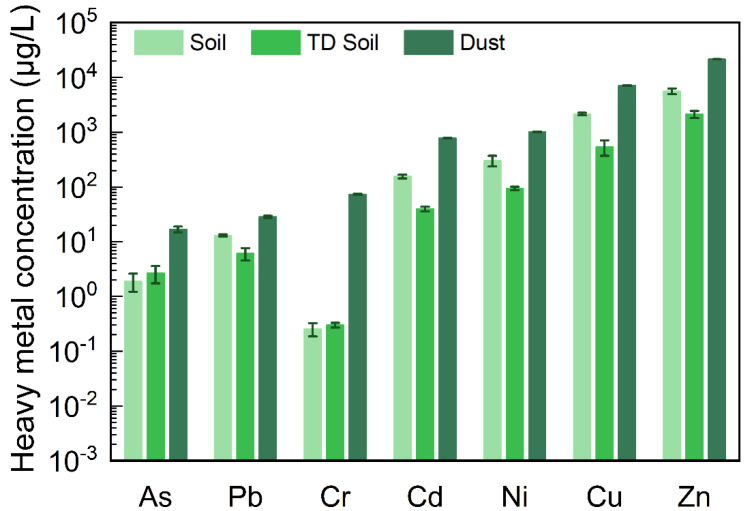
TCLP leaching concentrations of HMs in soil, TD soil and dust (*n* = 3). The leaching concentrations of HMs, from low to high, were in the following order: As, Pb, Cr, Cd, Ni, Cu, and Zn. In soil, thermal desorption soil, and dust, the leaching concentrations of As were 1.90 μg/L, 2.66 μg/L, and 16.94 μg/L, respectively; the leaching concentrations of Pb were 13.09 μg/L, 6.11 μg/L, and 28.53 μg/L, respectively; the leaching concentrations of Cr were 0.25 μg/L, 0.30 μg/L, and 73.54 μg/L, respectively; the leaching concentrations of Cd were 155.06 μg/L, 39.73 μg/L, and 782.83 μg/L, respectively; the leaching concentrations of Ni were 305.24 μg/L, 94.48 μg/L, and 1015.31 μg/L, respectively; the leaching concentrations of Cu were 2144.52 μg/L, 542.79 μg/L, and 7097.99 μg/L, respectively; and the leaching concentrations of Zn were 5599.72 μg/L, 2134.14 μg/L, and 21,592.35 μg/L, respectively.

If soil contains non-volatile materials, such as HMs, it is required to be transported to a landfill for disposal after thermal desorption [16]. Related studies have assessed the effects of HMs from coal fly ash in landfills on groundwater [17], but no such study has been performed on the effects of baghouse filter dust on groundwater. Therefore, we used the TCLP leaching method to further evaluate and analyze the leaching characteristics of soil before and after dust and thermal desorption in this disposal scenario. The leaching concentrations of the seven HMs in dust were significantly higher than those in soils and TD soils (Figure 2). Except for those of As and Cr, the leaching concentrations of the five other HMs in the soil, in the TD soil, and dust samples initially decreased and then increased. The characteristics of the leaching concentrations of As and Cr in soils after thermal desorption differed from those of the other five HMs. Cr and As, in the form of oxygen-containing anions, have been inferred to change differently from other cationic metals during the thermal process [18].

The regulatory level of the maximum concentration of contaminants for the toxicity characteristic of Cd was 1000 μg/L [19], whereas the leaching concentration of Cd found in the dust was close to 800 μg/L, a value close to the standard value for hazardous waste identification. Therefore, disposal of dust in baghouse filters is a critical issue.

**Figure 3:**
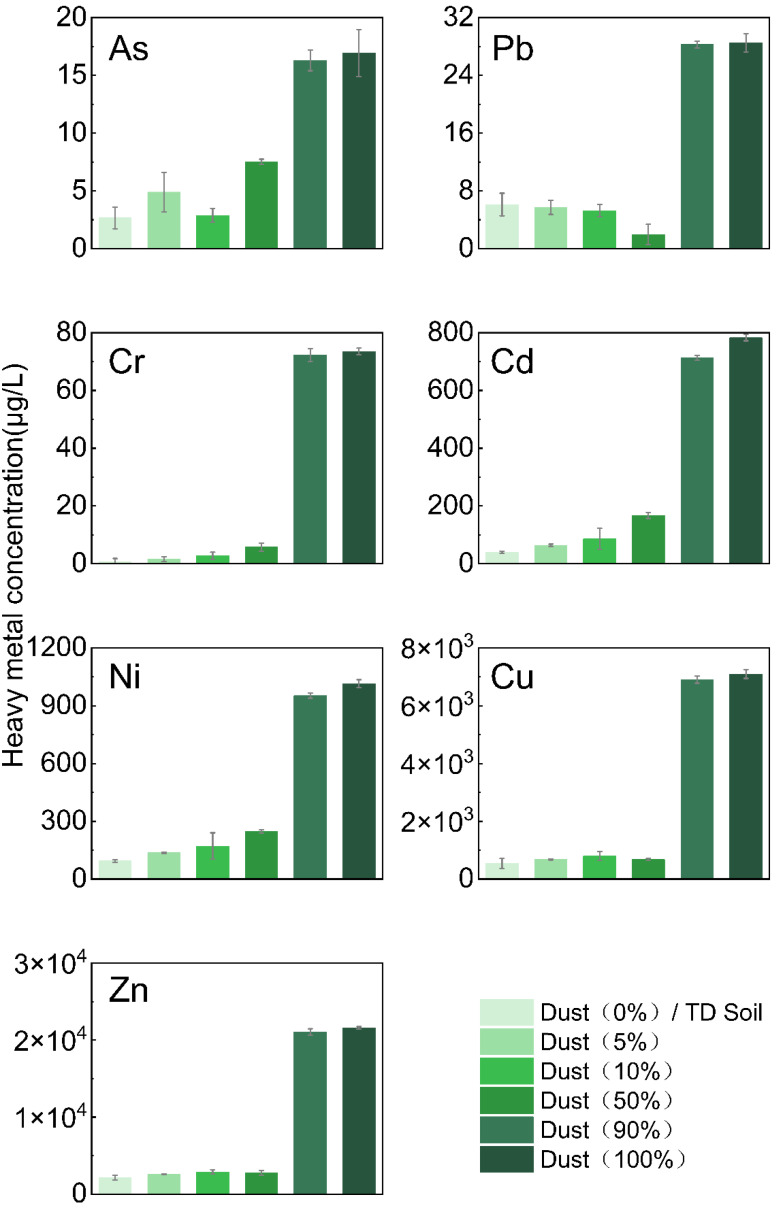
Leaching concentrations of seven heavy metals, determined by TCLP. The mass ratios of dust to the total mixed sample were 0%. 5%; 10%; 50%; 90%; and 100%. The leaching concentrations of heavy metals in dust, from low to high, were in the following order: As, Pb, Cr, Cd, Ni, Cu, and Zn.

In actual case sites, the general disposal method involves mixing dust with TD soil [20,21] (Figure S1g). In this study, the TCLP leaching concentrations of HMs in the mixed samples were measured after mixing the dust and TD soil samples in different proportions (SI), as shown in Figure 3. The results in Figure 2 and Figure 3 together suggested that the bag filter dust and TD soil should not be mixed, but instead should be separated and disposed of separately to decrease impacts on the environment. Perhaps the recycling of resources in this type of dust may be achieved in the future [22,23].

### 3.2. Bioaccessibility of HMs

**Figure 4:**
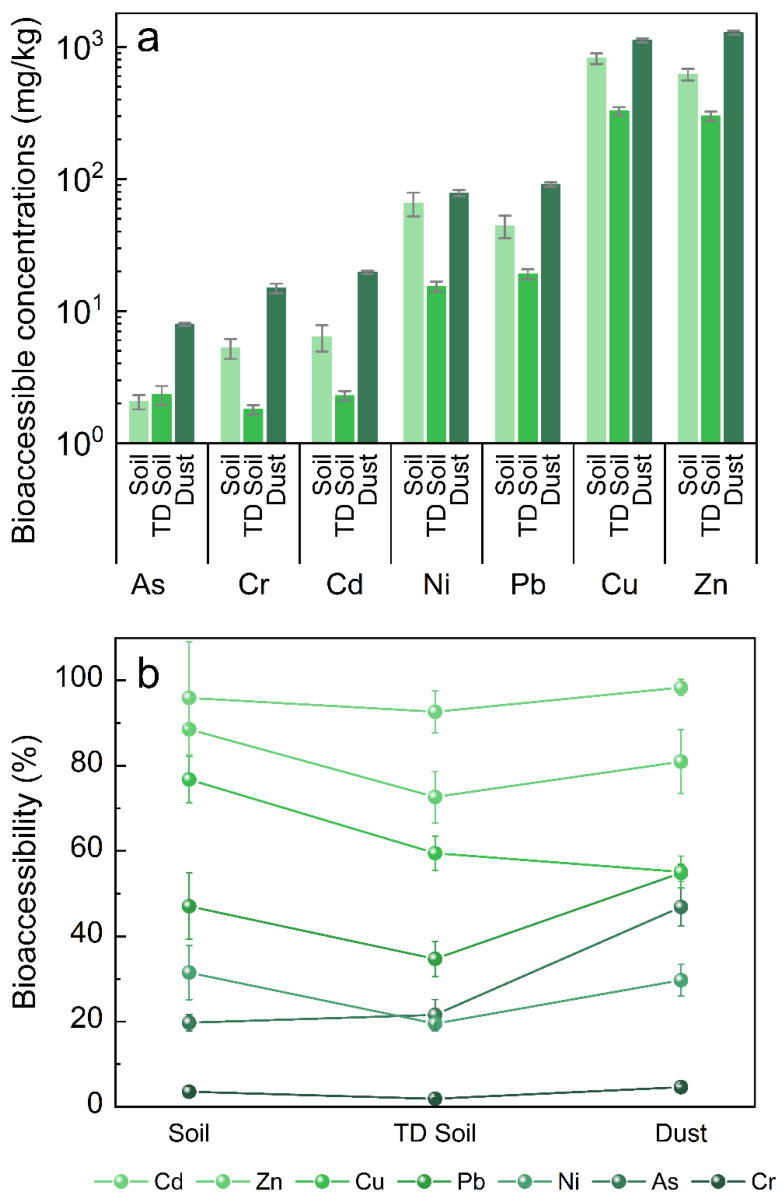
Bioaccessible concentrations (**a**) and bioaccessibility (**b**) of the seven HMs in different samples (*n* = 3). HMs are arranged in order of increasing bioaccessibility in dust. In soil, thermal desorption soil and dust, the bioaccessibility of Cr was 3.53%, 1.89%, and 4.61%, respectively; the bioaccessibility of Ni was 31.49%, 19.54%, and 29.7%, respectively; the bioaccessibility of As was 19.73%, 21.6%, and 46.9%, respectively; the bioaccessibility of Pb was 47.06%, 34.72%, and 54.89%, respectively; the bioaccessibility of Cu was 76.76%, 59.45%, and 55.08%, respectively; the bioaccessibility of Zn was 88.55%, 72.64%, and 80.97%, respectively; and the bioaccessibility of Cd was 95.94%, 92.67%, and 98.37%, respectively.

The concentrations of the seven bioavailable HMs were higher in the dust samples than the soil samples (Figure 4). However, not all HMs showed these trends: Cu was clearly lower in dust than soil, and Ni showed no difference. The bioaccessibility of Cr was low, at less than 5% in all samples. The bioavailable concentration of Cd was high, at nearly 100% in all samples. Because of the small particle size of dust, exposure and entry into organisms easily occurs through a variety of routes. The proportion of the Cd leaching concentration is noteworthy, and testing the bioaccessibility of HMs is useful in the risk quantification for dust [24].

The bioavailable concentrations of seven HMs in dust samples were significantly higher than those in the TD soil. Except for Cu, the bioaccessibility of HMs in dust samples tended to be higher than those in TD soil. Under the specific leaching conditions of SBET, the bioaccessible concentrations of Cu in both TD soil and dust were lower than those in the original soil during the thermal process, thus indicating different leaching characteristics from those of other metals because of different volatilization rates [25].

However, according to the SBET results, the thermal desorption rotary kiln itself has a stabilizing effect on most HMs [26]. Most HMs (except As) in the soil after thermal desorption showed both lower and weaker bioaccessibility than were observed in the original soil (Figure 4). Thus, although thermal desorption has many irreversible effects on soil physical and chemical properties, it does stabilize HMs. However, As, in contrast to other HMs in this process, was “activated” in the TD soil after thermal desorption in a rotary kiln; this finding is worthy of further exploration (Figure S2). In the thermal process, particularly in the secondary combustion chamber, the stability of As in the soil has been suggested to be greatly perturbed [27], thus “activating” As, which was originally stable in the lattice (Figure S2). Organic compound degradation might possibly affect these compounds’ mobility and availability [28,29]. It might be that high temperature changes the point of incorporation of organic matter with As, or changes the structure of arsenic-containing minerals, and further changes its REDOX potential after being dissolved in water, which may lead to the easier dissolution of As [30,31].

### 3.3. Relationship between Dust Particle Characteristics and Leaching Behavior

**Figure 5:**
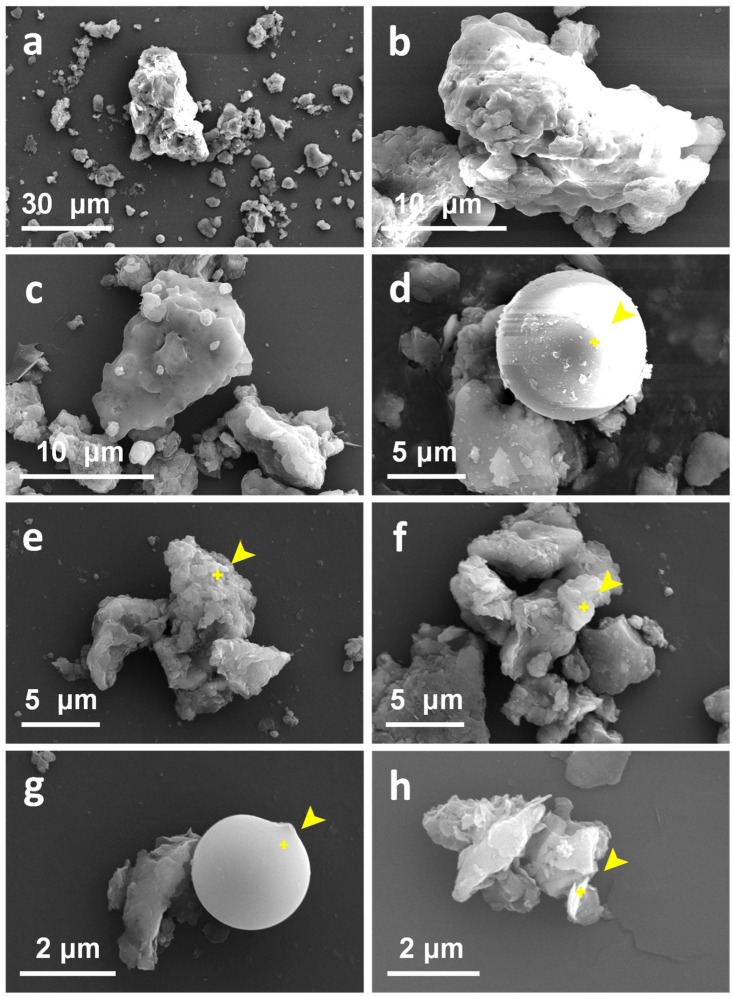
SEM images of baghouse filter dust particles. SEM images at ×1000 in (**a**); ×4000 in (**b**,**c**); ×5000 in (**d**–**f**); and ×15,000 in (**g**,**h**). The energy spectra of the points (marked in yellow) in (**d**–**h**) show the main component compositions of the particles: (**d**) O (50%), Si (19%), C (9%), Al (8%), and Ca (5%); (**e**) C (41%), O (30%), Si (23%), Al (2%), and Mg (1%); (**f**) O (48%), Si (22%), C (20%), Al (5%), and Na (4%); (**g**) O (50%), Si (26%), C (10%), Al (7%), and Fe (3%); and (**h**) Si (40%), O (34%), C (20%), Al (4%), and Fe (1%).

Figure 5 and Figure S3 show SEM images of baghouse filter dust. As indicated in Figure 5a (×1000) and Figure 5b,c (×4000), the sizes of the dust particles varied, and the shapes clearly differed. Analysis of the main components of particulate matter (Figure 5d–h) indicated that key element types were not substantially different from those in the soil. O, Si, and Al remained the main components, but the proportion of the main elements differed from those in the soil. In addition, the proportion of carbon elements was also relatively high in dust. Therefore, the differences in the leaching characteristics of HMs in dust may be due to the changes in the proportion and existing forms of the main components under the high temperatures of the rotary kiln and secondary combustion chamber.

As shown in Figure 5, the size of the dust particles was small. On the basis of these findings combined with previous experimental results, the particle size distribution of the dust samples was primarily within the 0–30 µm range [8]. Therefore, the HMs of soil particles with particle sizes in this range might have different leaching characteristics [32,33], thus causing high leaching concentrations in dust. Regardless of whether this finding was due to the smaller dust particles or to the high temperature destroying the original crystal structure or the organic composition, the leaching concentrations of HMs were higher in dust.

Some small particles with varying degrees of cohesion or agglomeration were observed (Figure 5). In the absence of agglomeration or cohesion, dust particles should theoretically pass through a 400-mesh (38 μm) sieve. However, in the actual operation process, when particle size dry sieving was performed, the dust particles passed through a 100-mesh (150 μm) sieve, whereas the mass proportion of the dust particles sieved by the 400-mesh sieve was very small. Therefore, a large cohesive force remained between the dust particles. The difference between dry sieving and particle size determination is that water is used as a dispersant in the particle size determination process, thus causing the force between the original particles to disappear or be weakened. Therefore, if the environment of the foundation pit is dry after backfilling of the dust, and the depth is higher than that of the groundwater, the process is relatively safe for the water environment. In contrast, if the water level of the groundwater is higher than the bottom of the foundation pit, the environmental risk increases: not only would the dust particles have a greater tendency to migrate toward the groundwater, owing to the weakening of the cohesion or electrostatic interaction, but the HMs within them would leach out in large quantities. The observed leaching concentration levels were much higher than those in the original organic contaminated soil and, therefore, might negatively affect the surrounding water environment.

## 4. Conclusions

Thermal desorption was developed for the treatment of various types of contaminated soils. However, limited data are available regarding the leaching characteristics of dust and TD soils from large-scale thermal desorption plants, thereby preventing a comprehensive understanding of the treatment process and the possible utilization of the products. Herein, risk analysis of dust was performed to assess the bioaccessibility and eco-toxicity of HMs. Furthermore, the environmental risk of dust in terms of the leaching behaviors of seven different HMs (As, Cd, Cu, Cr, Pb, Ni, and Zn) was estimated. The following conclusions were drawn:

(1) Compared with soil, dust had stronger leaching activity/transferability of HMs. Under several extraction methods, the extraction concentration of HMs in the dust of the newly identified baghouse filter dust, which should be treated as solid waste, showed a relatively high concentration of extraction phenomenon. This finding indicated that the pollution characteristics of HMs in baghouse filter dust are reflected by the total concentrations of HMs and high activity and bioaccessibility of heavy metal elements.
(2) The thermal process influences the stability of HMs in contaminated soil. The thermal desorption process may activate arsenic in soil. Both rotary kilns and secondary combustion chambers influence the existing forms of HMs and affect the leaching concentrations in different leaching environments, thus resulting in different leaching characteristics of HMs.
(3) Scientifically guided disposal of the solid waste generated after thermal desorption is recommended, and dust and soil after thermal desorption should be disposed of separately. The leaching concentrations of HMs were relatively higher in dust, possibly because of the smaller dust particles or the high temperature destroying the original crystal structure or the organic composition. Dust samples consisting of small particles should not be mixed with the soil after being thermally desorbed for unified disposal in subsequent treatment. Instead, treating baghouse filter dust and TD soil separately is more suitable for decreasing environmental risks.

## Data Availability

Not applicable.

## Supplementary Materials

The following supporting information can be downloaded at: https://www.mdpi.com/article/10.3390/ijerph192416504/s1. References [29,34,35] are cited in the Supplementary Materials.

## Nomenclature

HMs	Heavy Metals
TD soil	Thermal desorption soil
TCLP	Toxicity characteristic leaching procedure
SBET	Simple bioaccessibility extraction test
