# Supplementary material for: Leaching Characteristics of Heavy Metals in the Baghouse Filter Dust from Direct-Fired Thermal Desorption of Contaminated Soil
Source: Int J Environ Res Public Health. 2022 Dec 8;19(24):16504. doi: 10.3390/ijerph192416504 (PMC9778458; doi:10.3390/ijerph192416504)
Supplement: Supplementary file 1 [file ijerph-19-16504-s001.zip › ijerph-2059231-supplementary.pdf]

Supplementary Materials for

Article

Leaching Characteristics of Heavy Metals in the Baghouse Filter Dust from Direct-Fired Thermal Desorption of Contaminated Soil

Panpan Wang [1,2,3], Yunzhe Cao [3,*], Bin Yang [3], Huilong Luo [1,2], Tian Liang [1,2], Jingjing Yu [1,2], Aizhong Ding [1], Lina Wang [4], Huiying Li [3], Hanlin Cao [3], Fujun Ma [2], Qingbao Gu [2] and Fasheng Li [1,2,*]

[1] College of Water Sciences, Beijing Normal University, Beijing 100875, China
[2] State Key Laboratory of Environmental Criteria and Risk Assessment, Chinese Research Academy of Environmental Sciences, Beijing 100012, China
[3] Technical Centre for Soil, Agriculture and Rural Ecology and Environment, Ministry of Ecology and Environment, Beijing 100012, China
[4] School of Chemical and Environmental Engineering, China University of Mining and Technology, Beijing 100083, China
* Correspondence: caoyunzhe@tcare-mee.cn (Y.C.); lifs@craes.org.cn (F.L.)

Number of pages: 9

Number of tables: 2

Number of figures:3

Table S1. Concentrations of heavy metals in samples, mg/kg

	Soil	Thermal desorption soil	Dust
As	11.31	10.86	17.28
Cd	3.75	2.46	19.85
Cr	96.51	88.90	325.44
Cu	704.36	503.90	2032.27
Pb	69.53	63.38	163.97
Ni	92.36	84.79	264.77
Zn	498.22	400.70	1581.87

For determination of heavy metal concentrations, soil samples, were digested with a 5-mL mixture of HNO_3, $HClO_4$, and HF (1:1:3, v/v/v). The digestion was performed in polytetrafluoroethylene tubes. Each tube was placed in a graphite furnace digestion instrument (PT60, POLYTECH, Beijing, China), with two stages of working conditions (stage I: 150 °C, 8 h; stage II: addition of 1 mL HF, 200 °C, nearly dry). Subsequently, the concentrations of As, Pb, Ni, Cr, Zn, Cu, and Cd were determined with inductively coupled plasma mass spectrometry (ICP-MS) analysis (7900, Agilent, Santa Clara, California, United States).

Table S2. Indexes of groundwater quality, μg/L

	Type III groundwater quality standard values
As	10
Cd	5
Cr*	50
Cu	1000
Pb	10
Ni	20
Zn	1000

*Because the evaluation index of Cr in the groundwater standard is the standard value of Cr (VI), this value was used only as a reference.

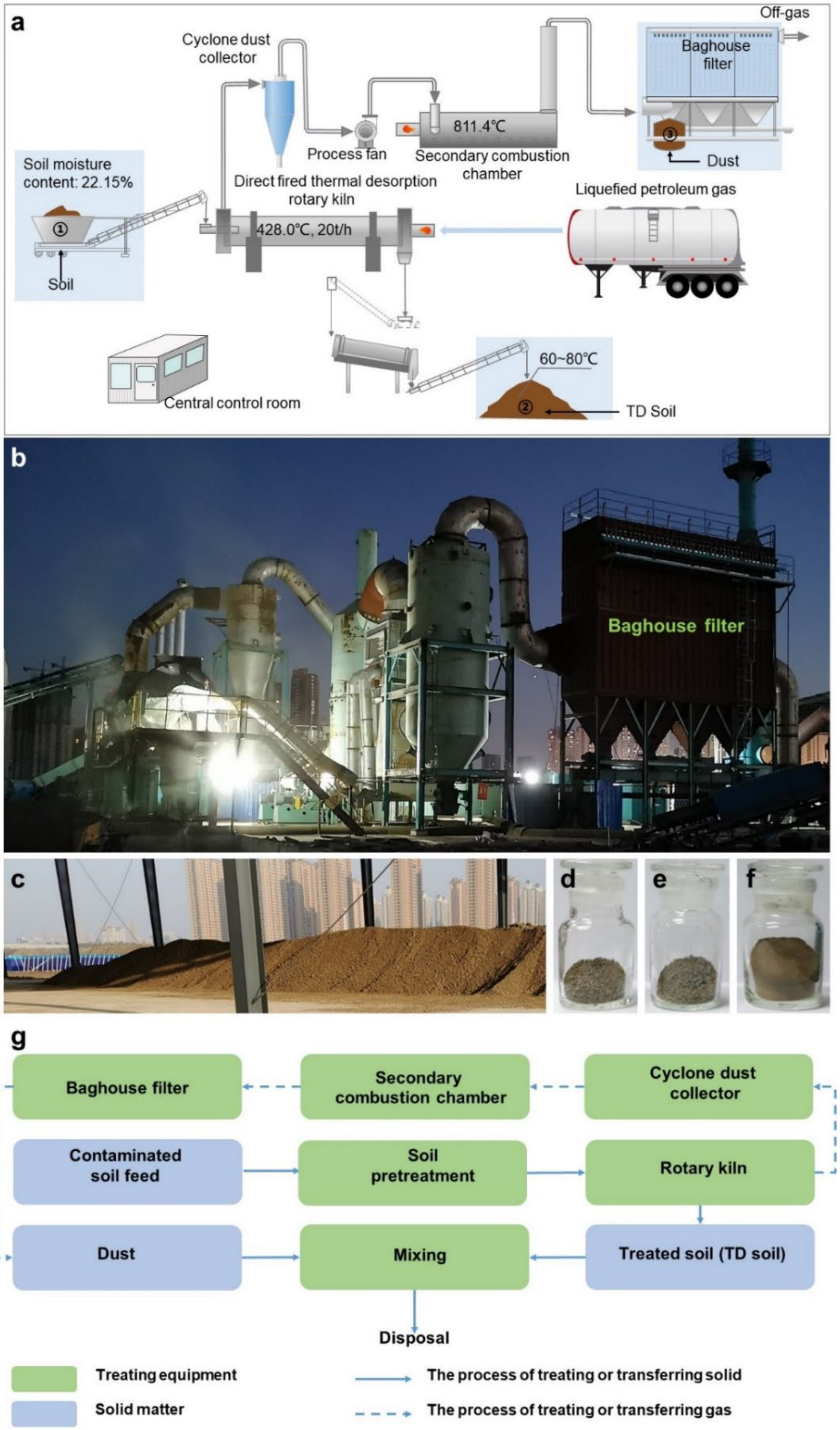

Figure S1. Flow chart of the entire process of ex-situ direct - fired thermal desorption

of contaminated soil from a thermal desorption disposal plant, including solid waste for disposal and soil samples collected. a: Flow chart of ex situ direct-fired thermal desorption at the site. The ex situ direct-fired thermal desorption consisted of a feed system, a desorption system, and a dust removal plant system. b: Ex situ direct-fired thermal desorption plant. c: Waste for disposal. d: Original soil sample passed through an 8-mesh (approximately 2 mm) sieve. e: Thermal desorption soil sample passed through an 8-mesh (approximately 2 mm) sieve. f: Dust sample; g: The final solid waste to be disposed of was a mixture of thermal desorption soil (treated soil) and baghouse filter dust. In this study, the original soil (contaminated soil), thermal desorption soil, and dust collected by the baghouse filter constituted the collected samples.

Sample mixing and testing According to the engineering data from the project, the amount of dust trapped in the baghouse filter was approximately 5% of the total amount of contaminated soil. However, an uneven engineering mixing process might potentially produce different leaching characteristics. To comprehensively consider this possibility, we studied six gradients of mixing ratios, with mass ratios of dust to mixed samples of 0%, 5%, 10%, 50%, 90%, and 100% (Fig.3). For preparation of mixed samples, the thermal desorption soil and dust samples were mixed in separate 2L PTFE tetrafluoroethylene bottles in different mass ratios. The mixed samples were shaken for 24 h (12 h forward rotation, 12 h reverse rotation) under a rotation speed of 30 rpm/min at room temperature of 25 °C. The mixed samples were temporarily placed in cold storage (4 °C). The procedure

for determination of mixed samples was as described in Section 2.3.

Speciation analysis of As

The Wenzel sequential extraction procedure was applied (Wenzel et al. 2001), and ICP-MS was used to determine the concentrations of the non-specifically-bound, specifically-bound, amorphous hydrous oxide-bound, crystalline hydrous oxide-bound, and residual phases. The extraction solutions and methods were as follows: I: 0.05 mol/L ammonium sulfate, 25 ℃, 4 h; II: 0.05 mol/L ammonium dihydrogen phosphate, 25 ℃, 16 h; III: 0.2 mol/L ammonium oxalate (pH=3), 25℃, 4 h; IV: 0.2 mol/L ammonium oxalate mixed with 0.1 mol/L ascorbic acid (pH=3), 96 ℃, 3 h; V: microwave digestion ($HCl:HNO_3$ = 3:1).The concentrations of As was determined with ICP-MS (7900, Agilent, Santa Clara, California, United States).

Correlation of As leaching behaviors with chemical fractionation

Given the particular leaching characteristics of As, we performed sequential extraction experiments. This sequential extraction was based on five fractions: non-specifically sorbed (F1), specifically sorbed (F2), amorphous and poorly-crystalline hydrous oxides of Fe and Al (F3), well-crystallized hydrous oxides of Fe and Al (F4), and residual phases (F5) [1].

Fig. S2 shows that the thermal processes, particularly the high-temperature stage of the secondary combustion chamber, destroyed the stable crystalline structure or binding state in the soil. The proportions of F1, F2, and F3 in dust were significantly higher than those in soil. The proportion of unstable chemical states in this particle size range (0–30

μm) might originally have been high [2], or the high-temperature process might have transformed the original stable chemical state of As in the soil to an unstable state [3].

The sequential extraction results were contacted with those of the above different leaching tests. The leaching concentrations of arsenic in the H_2O and TCLP leaching solutions were low, and the leaching concentrations of As in the sample fell within the range of the non-specifically sorbed fraction. Therefore, the proportion of As in the three sample types showed the same increasing trend as the non-specifically sorbed proportion.

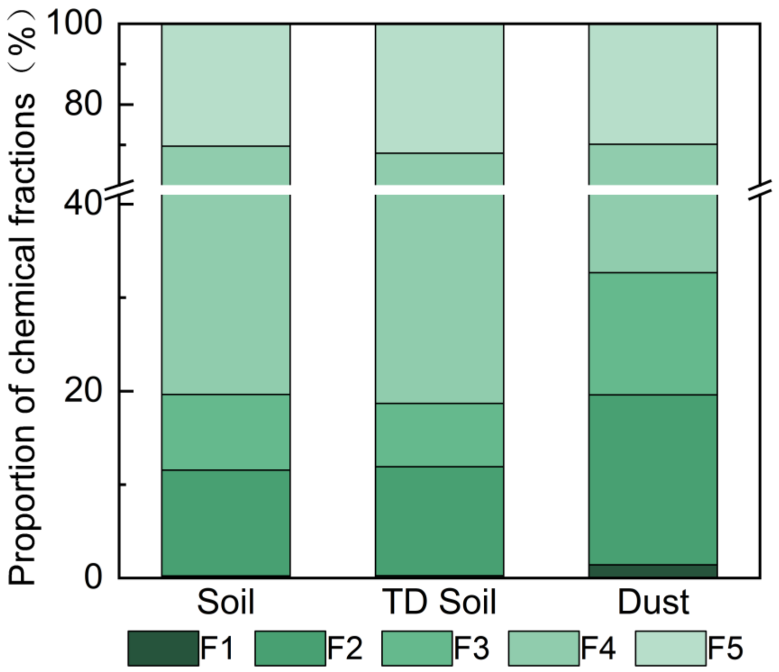

Figure S2. Percentages of total As concentrations in the five chemical fractions of three type of samples (n=3). Differences in non-specifically sorbed (F1), specifically sorbed (F2), amorphous and poorly crystalline hydrous oxides of Fe and Al (F3), well-crystallized hydrous oxides of Fe and Al (F4), and residual phases (F5) of As in the original soil, TD soil, and dust. The speciation analysis results in the soil were F1 (0.23%), F2(11.32%), F3 (8.09%), F4 (50.01%), and F5 (30.35%); those in the thermal desorption soil were F1 (0.25%), F2(11.66%), F3 (6.79%), F4(49.22%), and F5(32.08%); and those in the dust are F1 (1.42%),

F2(18.19%), F3 (13.06%), F4(37.47%), and F5(29.86%).

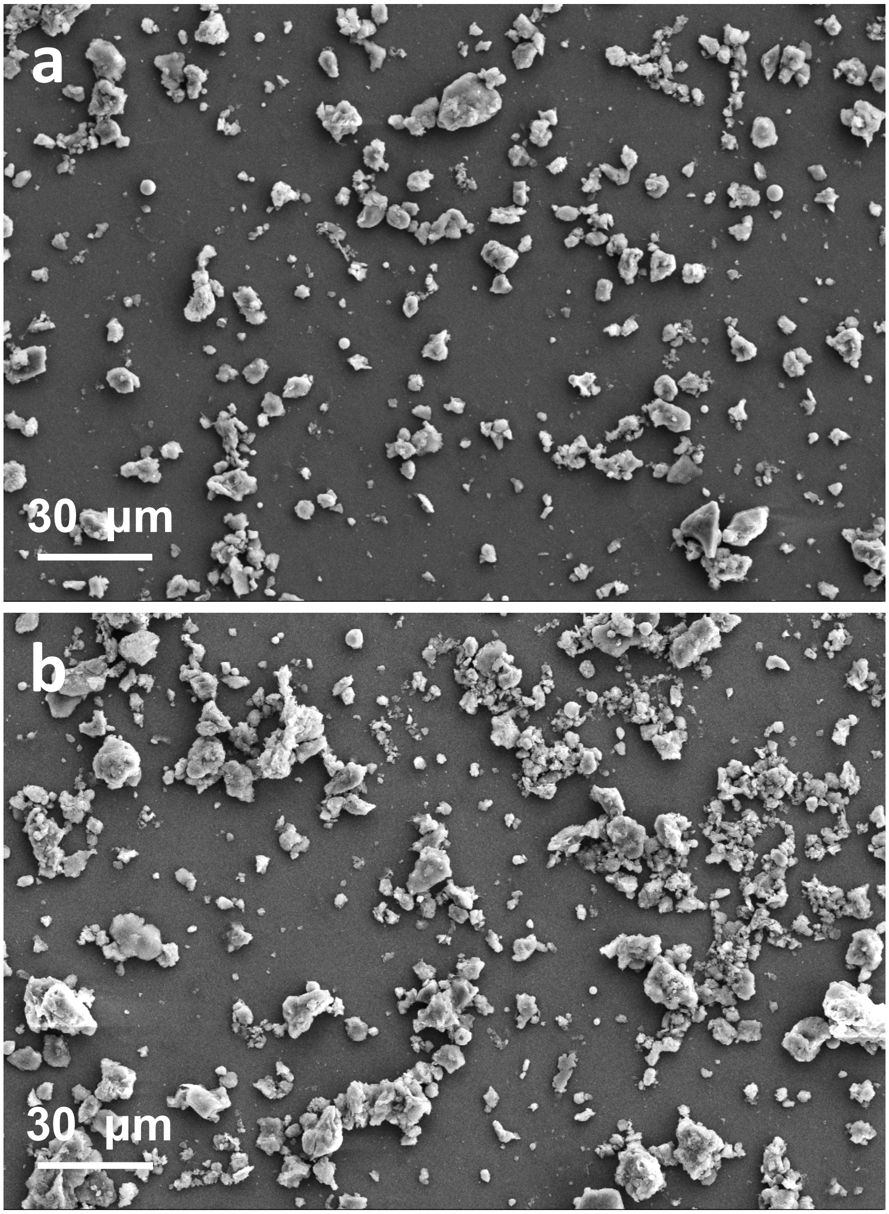

Figure S3. Scanning electron microscope (SEM) images of baghouse filter dust samples. Scanning electron microscope (SEM) images ×500 in (a), and ×500 in (b). (a): The samples were dispersed in anhydrous ethanol solvent by ultrasound and then dropped

onto a silicon wafer for drying. (b): The dried and ground samples were evenly dispersed on a silicon wafer, and the loose powder was blown off with a rubber suction bulb.

References

1. Wenzel WW, Kirchbaumer N, Prohaska T, Stingeder G, Lombi E, Adriano DC. Arsenic fractionation in soils using an improved sequential extraction procedure. Anal Chim Acta, 2001, 436, 309-323. https://doi.org/10.1016/S0003-2670(01)00924-2

2. Bernasconi D, Caviglia C, Destefanis E, Agostino A, Boero R, Marinoni N, Bonadiman C, Pavese A. Influence of speciation distribution and particle size on heavy metal leaching from MSWI fly ash. Waste Manage, 2022, 138, 318-327. https://doi.org/10.1016/j.wasman.2021.12.008

3. Su L, Wang S, Ji R, Zhuo G, Liu C, Chen M, Li H, Zhang L. New insight into the role of FDOM in heavy metal leaching behavior from MSWI bottom ash during accelerated weathering using fluorescence EEM-PARAFAC. Waste Manage, 2022, 144, 153-162. https://doi.org/10.1016/j.wasman.2022.03.023
